# COVID-19 vaccine hesitancy and acceptance in Mexico: a web-based nationwide survey

**DOI:** 10.26633/RPSP.2021.133

**Published:** 2021-10-18

**Authors:** Diego Ramonfaur, David Eugenio Hinojosa-González, Gloria Paulina Rodriguez-Gomez, David Alejandro Iruegas-Nuñez, Eduardo Flores-Villalba

**Affiliations:** 1 Harvard Medical School Cambridge United States of America Harvard Medical School, Cambridge, United States of America; 2 Instituto Tecnológico y de Estudios Superiores de Monterrey Monterrey Mexico Instituto Tecnológico y de Estudios Superiores de Monterrey, Monterrey, Mexico

**Keywords:** COVID-19, vaccination refusal, anti-vaccination movement, mass vaccination, surveys and questionnaires, Mexico, COVID-19, negativa a la vacunación, movimiento anti-vacunación, vacunación masiva, encuestas y cuestionarios, México, COVID-19, recusa de vacinação, movimento contra vacinação, vacinação em massa, inquéritos e questionários, México

## Abstract

**Objective.:**

To identify factors associated with COVID-19 vaccine hesitancy and acceptance among the Mexican population.

**Methods.:**

In a web-based nationwide survey in early December 2020, respondents were inquired about their sociodemographic characteristics and their willingness to accept a hypothetical COVID-19 vaccine given a 50% or 90% effectiveness. A logistic regression model was used to identify the factors associated with hesitancy and acceptance.

**Results.:**

A total 3 768 responses were analyzed. A 90% effective vaccine was accepted by 85% of respondents, while only 46% would accept being vaccinated with a 50% effective vaccine. In univariate analysis, each age group (40–49, 50–59, and ≥60) was strongly associated with vaccine hesitancy for a 90% effective vaccine (OR 0.48, 95% CI 0.38, 0.63; OR 0.33, 95 CI 0.26, 0.41; and OR 0.28, 95 CI 0.21, 0.38, respectively) compared to the 18–39 age group. After multivariable adjustment, similar magnitudes of association were observed. Being female and higher socioeconomic status were also associated with higher vaccine hesitancy.

**Conclusions.:**

Vaccine hesitancy represents a major public health problem in Mexico and is driven by multiple factors. Our study provides relevant insights for the development of effective policies and strategies to ensure widespread vaccination in Mexico.

## RESUMEN

**Objetivo.:**

Determinar los factores asociados a la aceptación y la reticencia frente a la vacunación contra la COVID-19 en la población mexicana.

**Métodos.:**

En una encuesta basada en la web a escala nacional, realizada a principios de diciembre del 2020, se preguntó a las personas entrevistadas sobre sus características sociodemográficas y su disposición a aceptar una hipotética vacuna contra la COVID-19 si garantizase 50% o 90% de efectividad. Se empleó un modelo de regresión logística para determinar los factores asociados con la aceptación y la reticencia.

**Resultados.:**

Se analizó un total de 3 768 respuestas. Una vacuna con 90% de efectividad fue aceptada por 85% de las personas entrevistadas, si bien solo 46% aceptaría una vacuna con 50% de efectividad. En el análisis con una variable, cada grupo etario (de 40 a 49, de 50 a 59 y más de 60) estuvo estrechamente relacionado con una reticencia a la vacunación de una vacuna con 90% de efectividad (OR 0,48, IC de 95% 0,38, 0,63; OR 0,33, IC de 95% 0,26, 0,41; y OR 0,28, IC de 95% 0,21, 0,38, respectivamente) en comparación con el grupo etario de 18 a 39 años. Tras el ajuste multivariante, se observaron magnitudes de asociación similares. También se asoció ser mujer y tener una mejor situación socioeconómica con una mayor reticencia a la vacunación.

**Conclusiones.:**

La reticencia frente a la vacunación representa un importante problema de salud pública en México y está impulsada por diversos factores. Nuestro estudio ofrece observaciones pertinentes para la elaboración de políticas y estrategias eficaces que garanticen una vacunación generalizada en México.

## RESUMO

**Objetivo.:**

Identificar os fatores associados a hesitação e aceitação em relação à vacina contra a COVID-19 na população mexicana.

**Métodos.:**

Em uma pesquisa nacional via internet, no início de dezembro de 2020, os participantes foram questionados acerca de suas características sociodemográficas e sua disposição de aceitar uma vacina hipotética contra a COVID-19, com eficácia de 50% ou 90%. Foi utilizado um modelo de regressão logística para identificar os fatores associados à hesitação e à aceitação.

**Resultados.:**

Foi analisado um total de 3.768 respostas. Uma vacina com eficácia de 90% seria aceita por 85% dos participantes, enquanto uma vacina com eficácia de 50% seria aceita por somente 46%. Na análise univariada, cada faixa etária (40-49, 50-59, e ≥60) apresentou forte associação com hesitação em relação a uma vacina com eficácia de 90% (OR 0,48, IC de 95% 0,38, 0,63; OR 0,33, IC 95% 0,26, 0,41; e OR 0,28, IC 95% 0,21, 0,38, respectivamente), em comparação com a faixa etária de 18-39 anos. Após ajuste na análise multivariada, magnitudes semelhantes de associação foram observadas. Ser do sexo feminino e de faixa socioeconômica de renda mais alta também mostrou associação com maior hesitação em relação à vacina.

**Conclusões.:**

A hesitação em relação à vacina representa um grande problema de saúde pública no México, e é impulsionada por múltiplos fatores. Nosso estudo traz informações relevantes para o desenvolvimento de políticas e estratégias efetivas para assegurar ampla vacinação no México.

As of March 2021, SARS-CoV-2, the virus that causes coronavirus disease 2019 (COVID-19), has reached over 116 million registered cases worldwide and caused more than 1.8 million deaths (1). This has resulted in tremendous economic, social, and political repercussions in virtually every country (2–3). Effectiveness of efforts to stop the spread of this virus has been blunted by poor compliance and civil disobedience; as a result, many countries have experienced secondary outbreaks after containment measures were lifted (4–7).

Mexico has been one of the hardest hit countries worldwide by the pandemic, with over 1.8 million confirmed cases and more than 150 000 deaths due to COVID-19 (1). Apart from these direct effects, Mexico has had economic setbacks; before the gradual economic reopening, more than a million formal jobs were lost in the country and more than 12 million people were no longer economically active, according to a national survey (8).

It is critical for countries to achieve herd immunity to mitigate the spread of the virus and subsequently end the pandemic (9). Consequently, unprecedented efforts have been made to develop a safe and effective vaccine against SARS-CoV-2, with some having received approval for emergency use (10). Nonetheless, several hurdles lie ahead; vaccine mass production, purchase, storage, distribution, and logistics will require thorough cooperation between governments and pharmaceutical companies. Ultimately, the general population will have the right to decide whether to receive the vaccine or not.

Vaccine hesitancy is defined by the World Health Organization (WHO) SAGE working group as, “The delay in acceptance or refusal of vaccination despite availability of vaccination services” (11). This describes a behavior in which individuals have varying degrees of suspicion against one or all vaccines, possibly resulting in delay of application or rejection of a vaccine (11). Factors associated with vaccine hesitancy can be categorized into: 1) vaccine-specific (safety and effectiveness); 2) contextual influences (information from the media, socioeconomic status, religion, and culture); and 3) individual/social group influences (perceived risk, perceived need for vaccination, and perceived effectiveness) (12). Prior studies have suggested the plausibility of data from search engine queries to detect epidemiological trends (13). This study attempts to employ a web-based application to increase the potential reach of this survey. Future studies could attempt to combine both approaches for greater insights into population behavior.

The objective of this study is to compare vaccine hesitancy and acceptance between age groups and identify the relevant factors associated with the acceptance and hesitancy toward a free COVID-19 vaccine, given a 50% and a 90% vaccine effectiveness in preventing disease. The results of this study may aid in the development of public health policy and education strategies to address vaccine hesitancy.

## MATERIALS AND METHODS

This study was conducted with prior review and exemption by the Internal Ethics Review Board and science committee with the study number P000498-FAV. It was performed in accordance with institutional regulations and in adherence to national regulations such as “Ley Mexicana General de Salud en Materia de Investigación, Artículo 17” (Mexican General Health Law) and abides by the norms established in the Helsinki Conference of 1964 as well as its revision in 2012. Consent to participate in the study was obtained upon opening the survey link, before proceeding to the survey. If respondents did not agree to participate, the survey did not proceed.

### Design and setting

At the time the survey was distributed, no approved vaccine was available; therefore, all questions were based on a hypothetical vaccine. Studies in other countries were previously conducted with a similar strategy and design (14). All data were collected through a survey using Google Forms from 4 to 11 December 2020. The self-administered questionnaire was in Spanish and included 29 multiple-choice items and one open answer item (age). All survey items were required to be answered in order for the response to be recorded, and the survey was answered anonymously. The survey was distributed using social media platforms by an unrestricted snowball strategy. Facebook, Instagram, Reddit, and WhatsApp were used to publicize and distribute the survey. Study volunteers aided in the distribution of the survey to other states of Mexico in order to increase generalizability. Inclusion criteria to answer the survey were being 18 years or older and being of Mexican nationality. A pilot survey was distributed to 15 people one week before launching the survey online to ensure the survey was clear and understandable. Items were selected based on relevant demographic information and findings in similar studies (14, 15).

### Study variables

The dependent variable was the respondent’s willingness to be vaccinated with a 50% and/or a 90% effective vaccine in two separate scenarios. Both hypothetical scenarios included free vaccines endorsed by the government. Both questions had a binary answer (“yes” or “no”). Independent variables included sociodemographic characteristics like age, gender, academic grade, occupation, religion, and monthly household income. Age was analyzed as a four-group categorical variable (18–39, 40–49, 50–59, and ≥60 years of age). The full list of study variables is summarized in Table 1.

Duplicate responses screening was performed by comparing each row (response) to the next 10 responses. Identical responses within a five-minute timeframe or within a range of 10 responses were considered duplicates and deleted.

**TABELA 1.** Study variables recorded from the web-based survey

**Study variables**	**Possible answers**
**Dependent variables**	
Willingness to be vaccinated with a 50% effective vaccine	Yes No
Willingness to be vaccinated with a 90% effective vaccine	Yes No
**Independent variables**	
Age	18–39 40–50 51–60 >60
Gender	Female Male
Academic grade^*^	Primary Secondary High school Professional Postgraduate
Occupation	Student Employee Unemployed Homemaker Company owner Informal job Healthcare worker
Religion^**^	Atheist Agnostic Jewish Catholic Christian Other
Monthly household income (Mex$)	Low (<5 000 and 5 000–10 000) Medium (10 000–30 000 and 30 000–50 000) High (50 000–100 000 to >200 000)
**Study variables**	**Possible answers**
Financial dependance	Yes No
Trusted countries for developing a COVID-19 vaccine ^***^	United Kingdom Germany United States Russia China Other
Private insurance	Yes No
Main source of information	Scientific journals News Family Friends Physician Social media YouTube
Vaccinated against influenza in the past three years	Yes No
Diagnosed with COVID-19 at any point in time	Yes No
Health status ^***^	Hypertension Diabetes Prediabetes Obesity Cancer Cirrhosis Lupus Rheumatoid Arthritis Epilepsy Myocardial or cerebral infarction Asthma Pulmonary Emphysema Chronic kidney disease Other None of the above
Overall health perception	Healthy Not healthy
Previous rejection of a vaccine due to fear of adverse effects	Yes No
History of severe adverse effects secondary to vaccine	Yes No
Smoking	Yes No
“If you get infected with COVID-19, do you think you would have …x”	Severe disease that will require hospitalization Mild disease
Knowing someone who had died from COVID-19	Yes No
Trust in government sanitary recommendations	Yes No
Belief of origin of the pandemic	Natural Artificial
Belief in facemask usage	Yes No
Taking supplement with the purpose of preventing COVID-19 infection	Yes No
Belief that the vaccine should be obligatory	Yes No
State of residency	Variable not included for analysis

**Notes:**

* Converted to binary variable: professional graduate or not;

** Converted to binary variable: catholic and not catholic;

*** Respondents could select multiple answers.

**Source:** Prepared by the authors from the study data.

### Statistical analysis

Frequency of events is described as number (percentage). A multivariable logistic regression model was used to assess significance among associations, employing the Omnibus Tests of Model Coefficients for model testing as well as Hosmer–Lemeshow test for data appropriateness. Results of regression models are expressed as unadjusted odds ratio (OR) and adjusted odds ratios (aOR). An alpha of <0.05 was adopted for statistical significance. No missing data were found within our variables of study. Statistical analysis was conducted using Stata-IC v.16.

## RESULTS

A total of 3 896 responses were recorded. After screening, 41 duplicates, 51 respondents who did not live in Mexico, and 36 respondents who identified as underage were discarded. The remaining 3 768 responses were analyzed.

### Respondent demographics

Overall median age was 30 (interquartile range 23–49). Respondents identified as male in 1 525 (40.4%) cases, and 2 940 (78.0%) had completed a professional degree. The most frequent occupation reported by respondents was “Employee,” by 1 106 (29.3%). We recorded responses from 30/31 states and 1/1 federal entities (Mexico City). Most respondents (41%) lived in the state of Nuevo León. The great majority were catholic, 2 798 (74%). Private insurance was reported by 2 149 (57.0%) of respondents, with 2 182 (57.9%) declaring some form of government-provided insurance, and 868 (23%) having both types of insurance. Any comorbidity was reported by 1 634 (41.9%) of respondents. The complete demographic data are summarized in Table 2.

### Vaccine acceptance

If a 50% effective vaccine was available, only 1 709 (46%) would accept to be vaccinated. This number increased to 3 211 (85%) if the offered vaccine was at least 90% effective. Only 545 (14.4%) were not willing to accept a vaccine, and 12 (0.3%) responded they would take a 50% but not a 90% effective vaccine.

### Acceptance by age group

**FIGURE 1.**
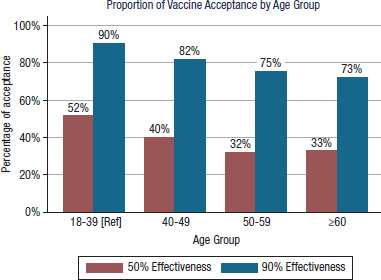
Vaccine acceptance for a 50% and a 90% effective vaccine, by age group

***Source*:** Prepared by the authors from the study data.

**FIGURE 2.**
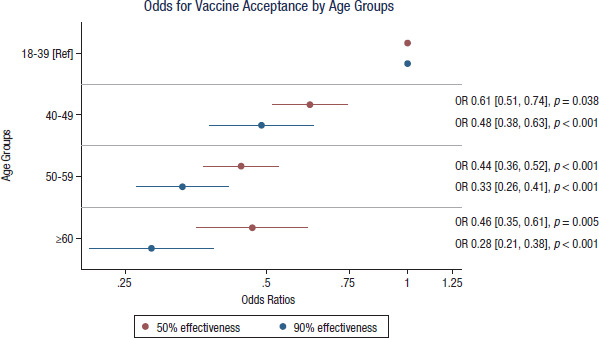
Odds ratios for acceptance and hesitancy toward a 50% and a 90% effective vaccine, by age group

***Note*:** Odds below 1 indicate hesitancy and odds above 1 indicate acceptance.

***Source*:** Prepared by the authors from the study data.

Age was divided into four categories, similar to the age groups proposed by the federal government according to their stepwise vaccination plan by age group. The youngest group was the largest, with 2 294 (58.8%) respondents, and was used as the reference group. The total number of respondents in the other groups were as follows: 584 (14.9%) in group 2, 629 (16.1%) in group 3, and 261 (6.6%) in group 4. A trend toward higher hesitancy was observed with older age groups, going from 90.2% to 72.4% acceptance in age groups 1 and 4, respectively, for a 90% effective vaccine (*p*-value for trend <0.001). The same pattern was observed for a 50% effective vaccine, going from 51.7% in the youngest group to 33.3% in the oldest group (*p*-value for trend <0.001) (Figure 1). In univariate analysis, we found age groups 2, 3, and 4 to be significantly more likely to reject the vaccine when compared to group 1 for both the 50% and the 90% effective vaccine (Figure 2). After multivariate adjustment for all variables in Table 2, similar magnitudes of association were observed among the age groups for a 50% effective vaccine (OR 1.02, 95% CI 0.65, 1.16; OR 0.48, 95% CI 0.36, 0.63; and OR; 0.32, 95% CI 0.22, 0.46; for each age group, respectively) and a 90% effective vaccine (OR 0.87, 95% CI 0.65, 1.16; OR 0.48, 95% CI 0.36, 0.63; and OR 0.32, 95% CI 0.22, 0.46; for each age group, respectively).

**TABELA 2.** Frequency and proportion of the total population with a positive variable, odds ratio and 95% confidence interval

	**Variable**	**Frequency (%)**	**Vaccine**	**Odds ratio**	**Low CI**	**High CI**	**p-value**
**Age**						
Group 1 (18–39) [Ref]	2 294 (60%)	50%	1				90%	1			
Group 2 (40–49)	584 (15%)	50%	0.61	0.51	0.74	0.038	90%	0.48	0.38	0.63	<0.001
Group 3 (50–59)	629 (16%)	50%	0.44	0.36	0.52	<0.001	90%	0.33	0.26	0.41	<0.001
Group 4 (≥60)	261 (7%)	50%	0.46	0.35	0.61	0.005	90%	0.28	0.21	0.38	<0.001
**Covariates**						
Male	1 525 (40%)	50%	1.41	1.24	1.62	<0.001	90%	1.42	1.17	1.72	<0.001
Professional degree	2 940 (78%)	50%	0.73	0.62	0.87	<0.001	90%	0.62	0.47	0.81	0.001
Economic independence	868 (23%)	50%	1.04	0.88	1.23	0.614	90%	1.04	0.84	1.29	0.694
Catholic religion	2 798 (74%)	50%	0.76	0.65	0.88	<0.001	90%	0.76	0.60	0.96	0.02
No trust of government	2 378 (63%)	50%	0.50	0.44	0.58	<0.001	90%	0.38	0.30	0.48	<0.001
High severity perception	359 (9%)	50%	1.15	0.92	1.44	0.202	90%	1.54	1.11	2.15	0.01
Previous COVID-19 infection	528 (14%)	50%	1.17	0.97	1.41	0.093	90%	1.25	0.94	1.65	0.121
Private insurance	2 149 (57%)	50%	0.64	0.56	0.73	<0.001	90%	0.59	0.49	0.72	<0.001
Any comorbidity	1 645 (43%)	50%	1.26	1.10	1.44	0.001	90%	1.77	1.46	2.16	<0.001
Smoker	1 404 (37%)	50%	1.10	0.96	1.25	1.161	90%	0.93	0.77	1.13	0.503
Living with someone older than 60	1 030 (27%)	50%	1.21	1.05	1.41	0.008	90%	1.33	1.07	1.65	0.008
Have rejected a vaccine before	835 (22%)	50%	0.23	0.19	0.27	<0.001	90%	0.25	0.21	0.30	<0.001
Have suffered vaccine adverse effects	248 (7%)	50%	0.48	0.36	0.64	<0.001	90%	0.50	0.37	0.69	<0.001
Influenza vaccine in the past three years	2 279 (60%)	50%	2.62	2.28	3.01	<0.001	90%	3.03	2.50	3.67	<0.001
Knowing someone who has died from COVID-19	2 868 (76%)	50%	1.04	0.89	1.21	0.586	90%	1.50	1.22	1.86	<0.001
Taking supplements to prevent COVID-19	1 235 (32%)	50%	0.64	0.55	0.74	<0.001	90%	0.79	0.65	0.95	0.017
**Primary source of information**						
News [Ref]	1 505 (40%)	50%	1				90%	1			
Scientific journals	666 (18%)	50%	1.66	1.37	2.01	<0.001	90%	1.26	0.93	1.71	0.131
Social media	859 (22%)	50%	0.81	0.68	0.97	0.021	90%	0.71	0.55	0.90	0.006
Friends and family	223 (6%)	50%	0.54	0.40	0.74	<0.001	90%	0.49	0.93	1.71	0.13
Physician	447 (12%)	50%	0.77	0.61	0.95	0.02	90%	0.66	0.50	0.88	0.005
YouTube	68 (1.8%)	50%	1.05	0.64	1.72	0.84	90%	0.36	0.20	0.65	<0.001
**Occupation**						
Student [Ref]	1 038 (27%)	50%	1				90%	1			
Employee	1 106 (30%)	50%	0.83	0.67	1.00	0.059	90%	0.65	0.46	0.91	0.12
Unemployed	77 (2%)	50%	0.91	0.56	1.27	0.702	90%	0.53	0.27	1.02	0.061
Company owner	547 (14%)	50%	0.59	0.46	0.76	<0.001	90%	0.42	0.29	0.61	<0.001
Informal job	201 (5%)	50%	0.67	0.48	0.94	0.02	90%	0.45	0.28	0.70	0.001
Homemaker	502 (13%)	50%	0.42	0.32	0.56	<0.001	90%	0.39	0.26	0.57	<0.001
Health worker	297 (8%)	50%	1.63	1.23	2.17	0.001	90%	2.05	1.13	3.72	0.018
**Household income**						
Low [Ref]	613 (16%)	50%	1				90%	1			
Middle	2 321 (61%)	50%	0.73	0.61	0.87	0.001	90%	0.78	0.58	1.04	0.102
High	833 (22%)	50%	0.56	0.45	0.69	<0.001	90%	0.47	0.34	0.64	<0.001

**Notes**: OR, odds ratio; [Ref], reference value.

ORs below 1 indicate hesitancy, ORs above 1 indicate acceptance. All covariates are adjusted for age.

**Source**: Prepared by the authors from the study data.

### Variables associated with vaccine hesitancy or acceptance

Prominent variables associated with a higher vaccine acceptance for both 50% and 90% effective vaccines included being male, having any comorbidity, living with someone older than 60 years of age, and having been vaccinated against influenza in the past three years. Having scientific journals as the primary source of information was only associated with a higher acceptance of a 50% effective vaccine when compared to the reference group (news). Variables associated with higher hesitancy for both 50% and 90% effective vaccines included having a professional degree, belonging to the middle- or high-income category, being catholic, having rejected a vaccine before due to fear, having had a serious adverse effect attributed to a previous vaccine, and taking supplements with the purpose of preventing COVID-19 infection. Having private insurance was only associated with rejecting a 50% effective vaccine. Complete results are summarized in Table 2.

### Other variables

When asked about the origin of the COVID-19 pandemic, 60% of participants answered they believed it arose from a natural origin, while 40% answered it was created as a bioweapon. Only three respondents (0.001%) answered they believed the pandemic is a hoax. Some 97% of respondents answered they believe facemasks should be routinely used; 69% believe everyone should be obligated to be vaccinated against COVID-19. When asked about the kind of test they would get to diagnose COVID-19 if symptomatic, 76% picked polymerase chain reaction (PCR) as their test of choice, 14% chose rapid antibody blood test, 8% answered “don’t know,” and 3% opted not to perform any diagnostic testing.

## DISCUSSION

Vaccination is a crucial intervention to mitigate the current COVID-19 pandemic. The design and manufacture of vaccines alone represents a significant challenge. Moreover, governments face important pitfalls like anti-vaccine movements, which have been mobilized globally and have resulted in outbreaks of virtually eradicated diseases like measles (16). Hesitancy toward the COVID-19 vaccine may cause setbacks to public health efforts attempting to ameliorate the pandemic. In our study, we seek to aid public health policymakers create a plan of action to improve targeted marketing strategies in an effort to combat hesitancy. The findings of this study offer important insights for government authorities regarding current vaccine hesitancy challenges, allowing for a more thorough planning of health care strategies and policy-making.

In line with studies conducted in other countries, our results indicate that the majority of respondents were willing to take a 90% effective vaccine (14). According to the most recent studies evaluating the efficacy of the Pfizer–BioNTech vaccine, an efficacy of more than 90% is estimated for most people currently receiving the vaccine (17). As a result, our study’s findings may be used to estimate the acceptance of vaccines with similar effectiveness. As for a 50% effective vaccine, only 46% of respondents were willing to accept this hypothetical vaccine. However, we speculate that the proportion of acceptability might be underestimated because by the time the survey was distributed, media announcements had recently been made regarding the >90% effectiveness of the Pfizer–BioNTech COVID-19 vaccine. A remarkable difference in the population studied compared to studies done in other countries is that our data suggest vaccine acceptance is higher among younger individuals. This association persisted even when adjusting for comorbidities and occupation. We believe this finding in our population may be explained by differences in access to and interpretation of information. Although the association of the second age group with hesitancy lost significance after adjusting for all the covariates in Table 2, our data still suggest a solid relationship between age and hesitancy in our study population.

Recently, the Grupo Técnico Asesor de Vacunación COVID-19 (COVID-19 technical advisory group) published a strategic plan of prioritization recommending prompt vaccination of high-risk groups (18). Our findings indicate that high-risk populations might not necessarily be more willing to take the vaccine compared to those with low risk. In particular, individuals in the oldest age group were significantly more likely to reject any vaccine regardless of its effectiveness. However, it must be taken into account that this population represents only 7% of the respondents. Moreover, even respondents who self-reported a higher perception of risk for severe disease were not more likely to accept the vaccine, compared to respondents who self-reported a perception of lower risk. Additionally, the acceptance of a 90% effective vaccine was lowest in the age group with the highest risk for disease progression. According to a recent phone survey deployed by the Mexican federal government and designed to evaluate vaccine acceptance among the older-adult population (>60 years), 71% of respondents agreed to be vaccinated (19), which is very similar to our survey’s 73% acceptance for a 90% effective vaccine. Additional data from the Ministry of Health (Secretaría de Salud) surveys reported an estimated 65%–75% acceptance rate in the surveyed population, with an estimated 70% for adults aged 50–59 years, similar to the 75% identified in our survey for the same age group. While our findings suggest a much higher overall acceptance rate (85%), this disparity could be attributable to a variety of factors, such as differences in reach, timing (December 2020 vs. April 2021), pandemic longevity, and increased acceptance rates in younger cohorts (20).

Our data strongly suggest that respondents with higher socioeconomic status tend to have higher prevalence of hesitancy. For example, having a professional degree, high income, or private insurance were strongly associated with vaccine hesitancy. These findings contrast with similar studies in other countries, where higher socioeconomic status was associated with a lower hesitancy. While the association we found may appear counterintuitive, the magnitude of association was robust among income, professional degree, and private insurance, even after adjustment for age. The reason for this association was not explored in our analysis, and the effect of unmeasured confounders cannot be ruled out. Similar to other studies, we found not having received influenza vaccine to be highly associated with hesitancy (21, 22). Distrust in federal government recommendations predominated among the respondents, with an overwhelming 63% reporting distrust of government. According to the global survey done by Lazarus et al. (22), government trust has a strong effect on vaccine rejection among respondents.

Both vaccine- and population-dependent factors were identified. Hesitancy for vaccines with low effectiveness is very high, while highly effective vaccines seem more promising to ensure widespread vaccination. Our work provides key insights for developing evidence-based interventions to increase vaccine acceptance among the population. While causality cannot be drawn from our study, it is in the best interest of federal governments to communicate effectively and transparently with the population in order to increase vaccine acceptance; for example, through public service announcements from nongovernmental organizations to address misinformation concerns in the population. Other interventions aimed at educating individuals regarding the safety and efficacy of vaccines may also play an important role in combating hesitancy. However, more research is needed to tailor these interventions. Another interesting phenomenon is the possible impact that emerging infectious diseases have on driving vaccine acceptance, as studies on non-emerging infections such as human papillomavirus have reported vaccine acceptance rates in the low 20% (23).

This is the first study in Mexico to evaluate COVID-19 vaccine hesitancy in a nationwide survey. Moreover, our work includes a thorough statistical analysis that allows for adequate comparisons between subgroups of the population.

Our study has a number of limitations. It is a cross-sectional study, which does not allow for drawing causal conclusions. Furthermore, this survey was distributed less than 48 hours after a >90% vaccine effectiveness was announced by pharmaceutical companies, which might have made respondents more hesitant toward the 50% effective vaccine option. For sanitary reasons, a web-based survey with a snowball sampling was the most reasonable way to conduct the study. As a result, we had less control over the demographic characteristics of the respondents, potentially introducing reporting bias, as we have little information about how many people—and for what reasons—decided not to answer the survey. Moreover, due to the web-based nature of the survey, our population is likely skewed toward younger individuals. Additionally, our sample had an overrepresentation of students, people with professional degrees, and people with higher socioeconomic status. On the other hand, because of the web-based nature of the survey, we speculate that people with limited access to electronic devices and social media are underrepresented in our survey. Due to the sampling approach used, this study relies on self-reported data, which may favor reporting bias. However, this limitation may be seen as an advantage, allowing for fully anonymous responses, which may reduce information suppression from respondents.

Effective nationwide vaccination is of paramount importance to mitigate the spread of COVID-19. Vaccine hesitancy may delay successful vaccination strategies, which would represent an important barrier toward widespread vaccination. Knowledge of the factors associated with vaccine acceptance or refusal may be helpful in developing vaccination policy. Vaccine acceptance among respondents in this study was highly influenced by vaccine effectiveness. There are many factors that influence vaccine hesitancy. Being familiar with these may allow for a more thorough vaccination strategy and policy development. We encourage health authorities to develop strategies aiming to increase vaccine acceptance among the Mexican adult population. Further studies exploring strategies to address acceptance and hesitancy of approved vaccines in Mexico will provide more information regarding this phenomenon, as more and more accurate information regarding vaccine efficacy and safety continues to be released.

## Disclaimer.

Authors hold sole responsibility for the views expressed in the manuscript, which may not necessarily reflect the opinion or policy of the *RPSP/PAJPH* or the Pan American Health Organization (PAHO).
